# High-efficiency ultra-thin CIGSe solar cells: defect engineering and back-surface field design

**DOI:** 10.1039/d6ra02870e

**Published:** 2026-06-08

**Authors:** Serap Yiğit Gezgin, M. A. Basyooni-M. Kabatas, Hamdi Şükür Kiliç

**Affiliations:** a Department of Physics, Faculty of Science, University of Selcuk 42031 Selcuklu Konya Turkey; b Department of Precision and Microsystems Engineering, Delft University of Technology Mekelweg 2 2628 CD Delft The Netherlands m.kabatas@tudelft.nl; c Institute of Nanotechnology (INT), Karlsruhe Institute of Technology (KIT) Kaiserstraße 12 76131 Karlsruhe Germany m.kabatas@kit.edu; d Department of Nanotechnology and Advanced Materials, Graduate School of Applied and Natural Science, Selçuk University Konya 42030 Turkey; e Department of Metallurgical and Materials Engineering, Faculty of Engineering, University of Dokuz Eylül İzmir Turkey

## Abstract

This study presents a comprehensive SCAPS-1D simulation of an ultra-thin CIGSe/CdS/i-ZnO/ITO solar cell with a 420 nm absorber layer, focusing on the influence of key physical parameters and back surface field engineering. The effects of acceptor doping density in CIGSe (*N*_a_ = 10^13^ to 10^18^ cm^−3^), interface defect density (*N*_i–t_ = 10^9^ to 10^18^ cm^−3^), bulk defect density (*N*_t_ = 10^12^ to 10^20^ cm^−3^), and electron affinity (*χ* = 4.35–4.65 eV) were systematically investigated. Increasing *N*_a_ significantly enhanced device performance by strengthening the internal electric field and increasing the carrier concentration, thereby improving *V*_oc_, fill factor, and efficiency. In contrast, elevated interface and bulk defect densities led to severe recombination losses and significant degradation of all photovoltaic parameters. Optimal band alignment was obtained at *χ* ≈ 4.35 eV, corresponding to a slight negative conduction-band offset that facilitates carrier transport and suppresses recombination. Recombination analysis showed stable performance of the radiative recombination coefficient over the range 10^−16^ to 10^−8^ cm^3^ s^−1^, while Auger recombination became dominant at coefficients above 10^−23^ cm^6^ s^−1^. Among the investigated back surface field layers, Cu_2_O provided the best performance due to its wide band gap (2.2 eV) and strong back-surface electric field, yielding a maximum simulated efficiency of ∼40.3% with *V*_oc_ = 0.817 V, *J*_sc_ = 30.03 mA cm^−2^, and FF = 82.88%. Capacitance–voltage and Mott–Schottky analyses revealed that capacitance increases from 57.6 to 109.9 nF cm^−2^ with increasing *N*_a_, and the built-in potential ranges from 0.80 to 1.32 V, confirming enhanced junction properties. These results provide practical guidelines for optimizing ultra-thin CIGSe solar cells through defect control, band alignment tuning, and back surface field design.

## Abstract 2

High-efficiency ultra-thin CIGSe solar cells are numerically designed by tuning absorber doping, defect densities, band alignment, and Cu_2_O back-surface fields to suppress recombination and boost simulated efficiency to ∼40%.

## Graphical abstract

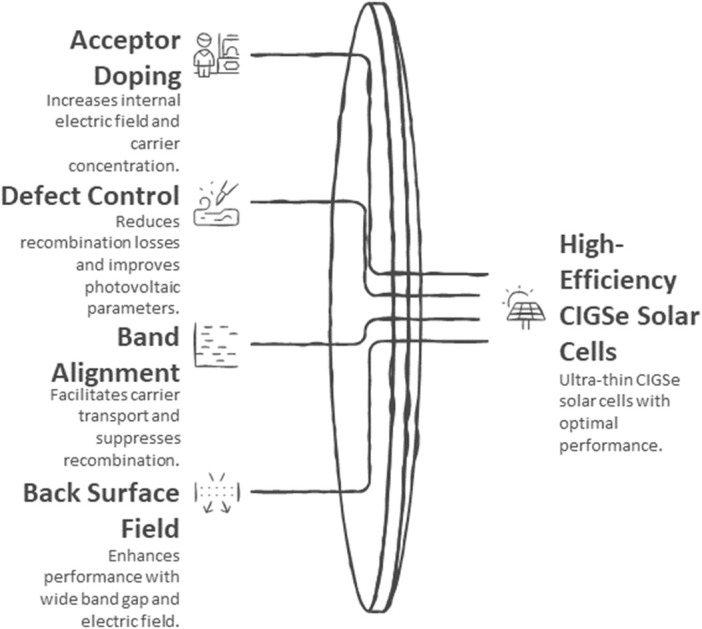

## 1 Introduction

Semiconductors belonging to the I–III–VI_2_ family, including CuInSe_2_ (CIS) and the gallium-alloyed CuIn_*x*_Ga_1−*x*_Se_2_ (CIGS), play a crucial role as light-absorbing layers in thin-film solar cells. CIGS has emerged as the leading material in the thin-film solar cell industry, distinguished by its exceptional light-harvesting capability, with an absorption coefficient exceeding 10^5^ cm^−1^, and its outstanding structural and environmental stability.^1^ The electronic band gap of CIGS can be tuned between 1.0 eV and 1.7 eV through variation of gallium content, “*x*”, in CuIn_1−*x*_Ga_*x*_Se_2_ composition, enabling precise control over its optoelectronic properties.^2^ The naturally limited abundance of indium (In) in CIGS compounds imposes a significant economic constraint, thereby increasing the production costs of thin-film solar cells that use CIGS as the primary absorber layer. To overcome this problem, the thickness of the absorber layer can be reduced to below 500 nm, and semiconductor layers of this thickness are referred to as ultra-thin films.^3^ Particularly in solar cell applications, ultra-thin films are favored to minimize material costs, facilitate the rapid transport of photogenerated electrons and holes to the electrodes, reduce the likelihood of carrier recombination, and ultimately improve device efficiency. In ultra-thin absorber layers, only a small fraction of photons is absorbed, limiting the generation of photoexcited charge carriers and reducing device performance. To address this issue, light-trapping techniques^2^ or back-surface field (BSF) layers are used to improve efficiency.^4^

BSF layers are heavily p^+^-doped and are placed between the back contact and p-type absorber layer, where they help support charge-carrier transport and improve device performance. By establishing a strong electric field at a p^+^–p junction, these layers guide minority carriers (electrons) from the p-type absorber toward the depletion region, reducing their recombination at the back contact and enabling more efficient carrier collection.^4^ Additionally, they enable the smooth transport of hole carriers from the absorber layer to the back contact, thereby improving overall charge collection. In solar cells, a variety of p^+^ type BSF materials such as SnS, V_2_O_5_, PbS, Sb_2_S_3_, Cu_2_O, CuO, and CuSCN, are widely utilized to assist carrier transport and contribute to improved device efficiency.^4–6^ Kumar *et al.* modelled a CIGS solar cell with CuAlO_2_, CuSbS_2_, FaSnI_3_, P_3_HT BSF layers and calculated efficiencies of 24.61%, 23.39%, 23.34%, 24.28%, respectively.^7^ In the different studies, Kumar *et al.* determined *J*_sc_ of 42.59 mA cm^−2^, 40.40 mA cm^−2^ and 35.64 mA cm^−2^, *V*_oc_ of 0.8 V, 0.92 V, and 0.99 V, FF of 80.38%, 83.34% and 87.91% and efficiency of 27.73%, 31.13%, and 31.08%, for a CIGS solar cell consisting of SCWT, Cu_2_O and CBTS BSF layers, respectively.^8–10^ Barman and Kalita achieved that efficiency of 24.22% with a 1.5 µm CIGS layer and a 0.15 µm PbS layer.^11^ Rahma *et al.* reported an efficiency of 21.835 for a CIGS solar cell with a 50 nm-thick Sn_2_S_3_ BSF layer.^12^ PV parameters of CIGS solar cell with CNGS BSF layer modeled by Oublal *et al.*, which are determined to be *V*_oc_ of 1.21 V, *J*_sc_ of 32.25 mA cm^−2^, FF of 75.08%, and efficiency of 29.39%.^13^

In calculating solar cell efficiency, the crystal, defect, electrical, and optical characteristics of each layer play an important role. The SCAPS-1D (Solar Cell Capacitance Simulator-1D) program enables the estimation of solar cell efficiencies based on the physical properties of the layers.^13,14^ Furthermore, the SCAPS-1D program is used to calculate the efficiencies of experimentally fabricated solar cells and to verify their performance. Photovoltaic (PV) parameters are determined by factors such as interface defects, band gap, film thickness, electron affinity, radiative recombination, and acceptor defect density, providing insight into how these variables influence solar cell performance.^15,16^

In this study, we modelled a solar cell using SCAPS-1D with a CIGSe absorber layer at 420 nm thickness that we previously produced using the PLD technique.^17^ We have calculated the potential efficiency of a solar cell by considering factors such as electron affinity, interface defect density, acceptor defect density, recombination mechanisms, and capacitance. Furthermore, the performance of the CIGSe ultra-thin film solar cell was significantly improved by using heavily p^+^-doped Sb_2_S_3_, CuO, and CuO_2_ BSF layers. *C*–*V*, *C*^−2^−*V*, and *C*−*f* characteristics were obtained as a function of the acceptor defect density. As a result, our findings provide insight into the expected performance of a CIGSe ultra-thin film solar cell with a thickness of 420 nm during experimental production.

## 2 The modeling of Au/BSF (Sb_2_S_3_, CuO, Cu_2_O)/CIGSe/CdS/i-ZnO/ITO solar cell

Numerical simulations play a crucial role in elucidating the physical characteristics and optimizing the design of solar cells. The advancement of simulation techniques has enabled comprehensive pre-experimental analysis and optimization of solar cell efficiency and structural design. This work utilizes the SCAPS-1D simulation platform to investigate the performance of the CIGSe solar cell. The software, developed at ELIS, Ghent University, is a one-dimensional solar cell simulator available for researchers worldwide. SCAPS-1D enables the grading of a wide range of physical parameters, such as band gap energy, acceptor density, electron affinity, interface defect density, operating temperature, serial and shunt resistance, *etc.*^17–19^

**Fig. 1:**
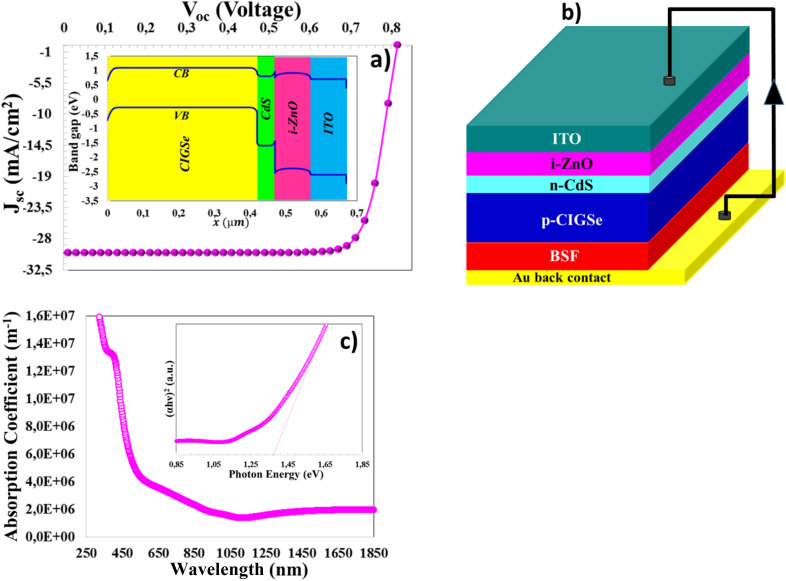
(a) *J*–*V* characteristic (band diagram in insert square), and (b) the schematic view of CIGSe/CdS solar cell with Cu_2_O BFS layer.

A numerical model of Au/CIGSe/CdS/i-ZnO/ITO solar cell configuration was developed in SCAPS-1D (Fig. 1(b)), based on experimentally validated parameters obtained from our earlier investigation of a monocrystalline CIGSe absorber layer.^17^ We predicted the PV response of the CIGSe solar cell model by analyzing the impact of varying defect-related, structural, and optical parameters, including *N*_i–t_, *N*_a_, *χ*_e_, and *B*_r_, *etc.* CIGSe semiconductor parameters derived from experimental measurements are given in Table 1, and the absorption coefficient spectrum of the ultra-thin film given in Fig. 1(c) was used as an input dataset in the simulation. The thin film thickness was measured at 420 nm, the band gap was determined from the Tauc plot in the small square shown in Fig. 1(c) as 1.37 eV, and carrier mobility was measured using the four-probe Hall effect. The physical parameters of all layers forming the solar cell were taken from the literature.^4,5,17,20,21^ PV parameters were calculated under illumination conditions of 100 mW cm^−2^. The *J*–*V* characteristic and band gap of the ideal solar cell modeled without BSF are given in Fig. 1(a). Sb_2_S_3_, CuO, and C_2_O BSF layers were used to further increase the efficiency of a thin absorber solar cell.

**Table 1:** Physical characteristics of the layers in the simulated Cu_2_ZnSnSe_4_ solar cell

Layers for solar cells	ITO^21^	i-ZnO^18^	CdS^20^	CIGSe^17^	p^+^Sb_2_S_3_^5^	p^+^ CuO^4^	p^+^ Cu_2_O^4^
Energy gap (eV)	3.3	3.3	2.4	1.37 (exp.)	1.62	1.51	2.2
Affinity of electron (eV)	4.6	4.6	4.2	Variable	3.7	4.07	3.2
The permittivity of dielectric	9	9	10	8.6	7.08	18.1	7.1
CB effective density (cm^−3^)	2.2 × 10^18^	2.2 × 10^18^	2.2 × 10^18^	7.91 × 10^17^	2.0 × 10^19^	2.2 × 10^19^	2.5 × 10^18^
VB effective density (cm^−3^)	1.8 × 10^19^	1.8 × 10^19^	1.8 × 10^19^	4.5 × 10^18^	1 × 10^19^	5.5 × 10^20^	1.5 × 10^19^
Thermal velocity of electron/hole (cm s^−1^)	1.0 × 10^7^	1.0 × 10^7^	1.0 × 10^7^	1.0 × 10^7^	1.0 × 10^7^	1.0 × 10^7^	1.0 × 10^7^
Mobility of electron/hole (cm^2^ V^−1^ s^−1^)	100/25	100/25	100/25	40/10 (exp.)	9.8/10	100/0.1	200/800
Density of shallow donor (cm^−3^)	1.0 × 10^20^	1.0 × 10^5^	1.0 × 10^18^	0	0	0	0
Density of shallow acceptor (cm^−3^)	0	0	0	Variable	1.0 × 10^15^	1.0 × 10^18^	1.0 × 10^18^
Thickness of film (nm)	100	100	50	420 (exp.)	Variable	Variable	Variable

In SCAPS, simulations can include as many as seven semiconductor layers, while the software solves the Poisson equation and carrier continuity equations1
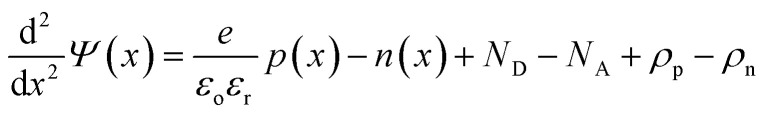
2
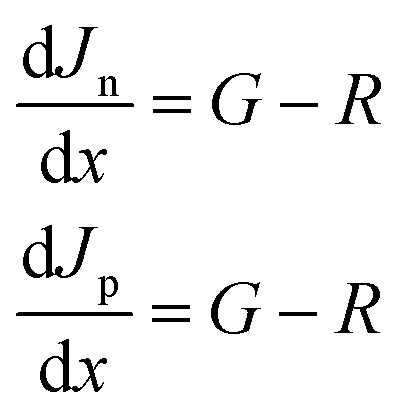
for electrons and holes.^17,22^ Where the parameters represented in these equations can be defined as below: *Ψ*(*x*) defines the electrostatic potential, *e* is the elementary charge, *ε*_r_ and *ε*_0_ denote relative and vacuum permittivity, *p* and *n* represent hole and electron concentrations, *N*_D_ and *N*_A_ indicate donor and acceptor impurity levels, and *ρ*_p_ and *ρ*_n_ describe hole and electron carrier distributions, *R* indicates the recombination rate, *G* represent generation rate.

The transport of electrons and holes in semiconductors is facilitated by drift and diffusion, which can be quantitatively described by eqn (3):3
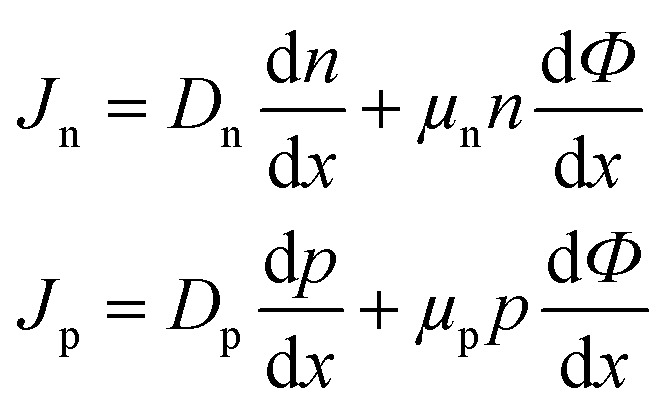


### 2.1. The impact of the acceptor defect density (*N*_a_) of the absorber layer

**Fig. 2:**
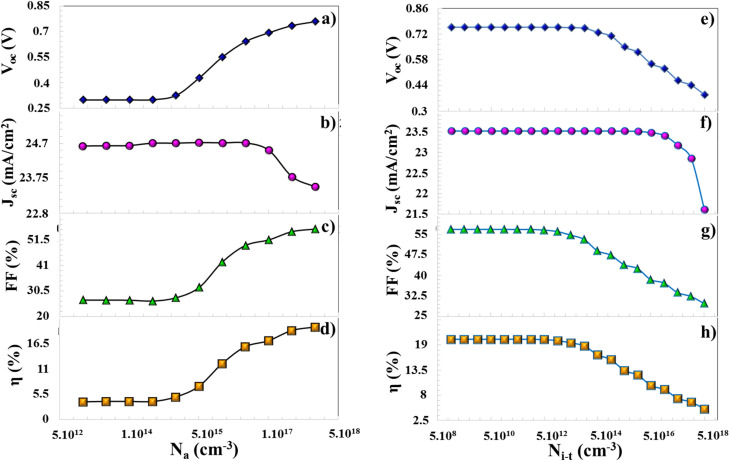
Variation of PV parameters: *V*_oc_, *J*_sc_, FF, *η* as a function of (a–d) *N*_a_ (cm^−3^) and (e–h) *N*_i−t_ (cm^−3^).

The acceptor carrier density (*N*_a_) of the CIGSe absorber layer significantly affects the solar cell's efficiency. Optimization of the absorber doping level can be achieved through the strategic increase of acceptor defect density.^23,24^ Enhanced doping levels increase the carrier density and the photogenerated carrier population, while defects introduce localized energy states that trap carriers; acceptor defects facilitate efficient carrier transfer. Introducing energy barriers for holes enhances carrier transport over long distances, while low-energy defect states act to form internal electric fields that guide carriers and reduce recombination losses,^25^ A rise in acceptor defect density leads to higher saturation current levels and impacts *V*_oc_, as illustrated in eqn (5), where the role of *N*_a_ reflects p-type doping influence:^26^5
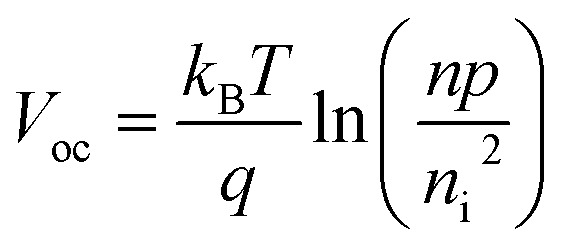
here, *T* indicates absolute temperature, *q* denotes the electron charge, and *k*_B_ represents the Boltzmann constant, while *n* and *p* correspond to the densities of photo-generated carriers and *n*_i_ defines the intrinsic carrier density. Higher *N*_a_ content in CIGSe thin film increases hole concentration, thereby enhancing carrier density and promoting charge accumulation in the depletion region. Increasing *N*_a_ concentration from 1 × 10^13^ cm^−3^ to 1 × 10^18^ cm^−3^^27^ resulted in considerable improvements in *V*_oc_, FF, and efficiency, which rose from 0.298 V to 0.757 V, 26.78% to 55.97%, and 3.835% to 19.953%, respectively, as depicted in Fig. 2(a–d). This increase can be attributed to the higher hole density resulting from increased p-type doping levels, the strengthened internal electric field, and decreased carrier recombination. The increased acceptor density facilitated the collection of photo-generated carriers by enabling more efficient charge separation in the hole region. High *N*_a_ values increased band bending, reducing recombination losses in the back-contact direction for carriers and supporting charge transfer. However, the fact that the performance increase approached saturation at very high doping levels indicates that excessive defect density can limit carrier lifetime by forming recombination centers. In CIGSe thin films, acceptor-type defects are not expected to be distributed at a single energy level or in a homogeneous manner; instead, there is a spatially heterogeneous distribution of different energy levels (shallow and deep) in intragranular, grain boundary, and interface regions. The presence of deep-level traps can negatively impact carrier lifetime by creating additional Shockley–Read–Hall (SRH) recombination centers, partially offsetting the positive effect of increased doping.

### 2.2. The impact of the interface defect density (*N*_i–t_) between CIGSe and CdS semiconductors

The formation of defect-induced energy levels at the interface affects how electrons and holes move across the absorber/buffer heterojunction.^28^ At the interface, fundamental defects such as dangling bonds, vacancies, and interfacial states can form, leading to electron–hole recombination and perturbing the local electric field, while fabrication-related issues, such as thin-film deposition, epitaxial growth, and band mismatch, also contribute to defect formation. In thin-film solar cells, interface defects generally do not exhibit a single-level, homogeneous structure; instead, they display a spatially heterogeneous distribution of multi-level trap structures with shallow and deep energy levels. While low and medium-level interface defects can support charge transfer by regulating band alignment in certain situations, excessive defect density leads to the dominance of deep-level trap centers. These deep-level conditions strengthen the SRH recombination mechanism, thereby increasing the probability of interface electron–hole recombination. Furthermore, the non-homogeneous defect distribution creates local potential fluctuations at the absorber/buffer interface, limiting carrier transport and increasing the series resistance effect. The impact of interface defects is minimal within the *N*_i–t_ range of 1 × 10^9^ to 1 × 10^13^ cm^−3^, indicating that interface recombination is negligible and carrier transport occurs efficiently. In this region, the internal electric field generated along the heterojunction is conserved, and photo-generated carriers can be collected with minimal loss. However, increasing the defect density (from 5 × 10^13^ cm^−3^ to 5 × 10^18^ cm^−3^)^29^ beyond this range significantly deteriorates PV parameters, the values of *V*_oc_, *J*_sc_, FF, and efficiency decreased significantly, dropping from 0.757 V to 0.389 V, 23.52 mA cm^−2^ to 21.60 mA cm^−2^, 56.54% to 29.78%, and 20.16% to 5.00%, respectively, as shown in Fig. 2(e–h). Increased defect levels at the interface cause electrons and holes to recombine there, forming SRH recombination centers. The significant decrease in *V*_oc_, in particular, is associated with a reduction in the carrier semi-Fermi level separation and an increase in the saturation current. The substantial decrease in FF can be attributed to increased charge-transfer resistance at the interface and decreased carrier-collection efficiency.

### 2.3. Impact of the conduction band offset (CBO)

The conduction band offset describes the energy difference between the conduction band minima of two semiconductors forming a heterojunction. Differences in electron affinity (*χ*) between semiconductor materials lead to conduction and valence band offsets, identified as CBO (Δ*E*_C_) and VBO, which are described in eqn (6) and (7):^22^

6 CBO = Δ*E*_C_ = *χ*_absorber_ − *χ*_buffer_

7 VBO = (*χ*_absorber_ + *E*_g absorber_) − (*χ*_buffer_ + *E*_g buffer_)

The interfacial energy barrier between absorber and buffer layers significantly impacts carrier transport mechanisms. A positive conduction band offset (*χ*_abs_ > *χ*_buff_) results in a spike-like barrier, which leads to recombination while restricting electron flow.^30^ To cross the heterojunction interface, photogenerated electrons must use their kinetic energy to surmount the existing potential barrier. For *χ*_abs_ < *χ*_buff_, the resulting negative CBO induces a cliff-type band configuration, associated with a reduced band discontinuity. As a result, photogenerated electrons can effectively surmount the interface barrier by utilizing their kinetic energy.

**Fig. 3:**
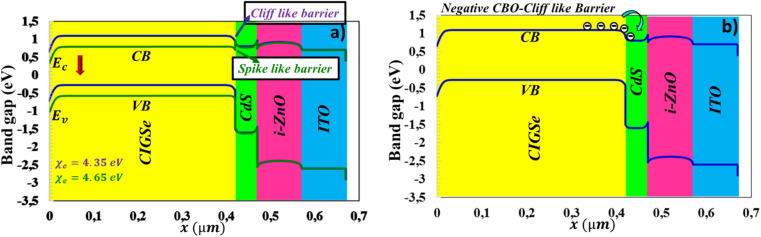
(a) A comparative band alignment illustrating cliff-type and spike-type interfacial barriers, and (b) a negatively offset cliff-like barrier configuration.

**Fig. 4:**
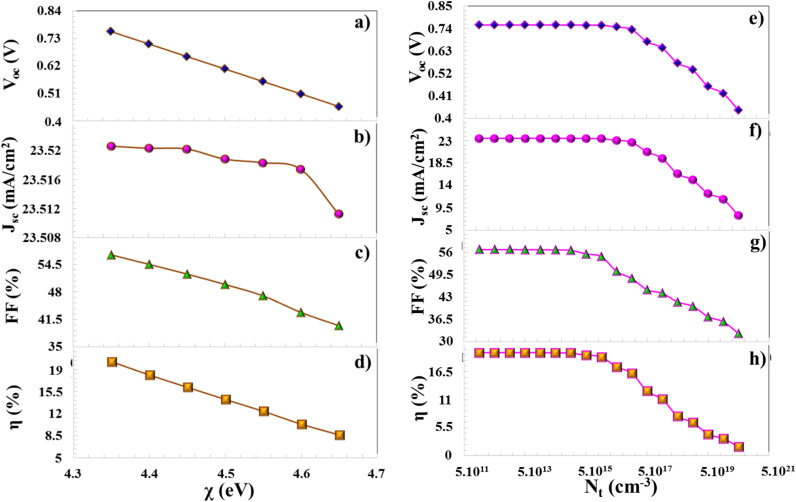
Variation of PV parameters: *V*_oc_, *J*_sc_, FF, *η* as a function of (a–d) *χ*_e_ electron affinity (eV) and (e–h) *N*_t_ (cm^−3^).

In the current study, device performance was analyzed as a function of the CIGSe electron affinity over the range *χ* = 4.35–4.65 eV.^16,31^ Elevating the electron affinity of CIGSe from *χ* = 4.35 eV to 4.65 eV leads to the formation of a spike-like conduction band offset at the absorber–buffer interface^32^ (Fig. 3(a)), impeding electron migration to CdS and promoting interfacial recombination losses. Spike-like (positive CBO) conduction band alignment creates an energy barrier for electrons at the absorber/buffer interface. In suitable (small and controlled) spike structures, this barrier can reduce interface recombination by restricting reverse carrier flow and can, in particular, increase *V*_oc_. It also improves carrier selectivity and enhances diode quality by preventing uncontrolled electron passage across the interface. Furthermore, high spike values can slow down carrier extraction, increasing the series resistance effect. Conversion: the creation of an energy barrier that prevents electron transport at the interface. As the barrier height increases, it becomes more difficult for photogenerated electrons to pass into the buffer layer, leading to carrier accumulation at the interface and increased SRH recombination. A cliff-type band alignment corresponding to Δ*E*_c_ = −0.05 eV is formed at *χ* = 4.35 eV (Fig. 3(b)). The observed negative valence band offset (Δ*E*_v_ = −1.08 eV) promotes a cliff-type band configuration that effectively suppresses the recombination of photo-excited carriers.^33^ The cliff structure formed at slightly negative CBO values can reduce the band discontinuity between the absorber and buffer layers, providing a more favorable energy alignment at the interface. This can accelerate charge transfer by reducing carrier accumulation in some devices and by helping maintain the occupancy factor (FF). It has been reported that small negative or near-zero CBO values can create an optimal balance between electron transport and interface recombination. However, the cliff-type conduction-band alignment, while facilitating electron transport, leads to electron accumulation at the interface at excessively negative potentials (a deep cliff structure), increasing SRH recombination and causing *V*_oc_ losses. It can also weaken carrier selectivity, increasing reverse current and diode losses. The effect is more complex due to the non-homogeneity of interface defects in the device and the presence of multi-level energy states. Grain boundaries and chemical irregularities enhance carrier localization and recombination. Therefore, cliff-tip alignment can limit performance, especially at large negative CBO values. At an electron affinity of *χ* = 4.65 eV, *V*_oc_, *J*_sc_, FF, and conversion efficiency drop to 0.458 V, 23.51 mA cm^−2^, 40.03%, and 8.62%, respectively, highlighting the performance deterioration (Fig. 4(a–d)).

### 2.4. The generation and recombination mechanism

#### 2.4.1. The impact of the defect density (*N*_t_) in the absorber semiconductor

In CIGSe thin-film absorber layers, the defect structure is not ideal or single-level; instead, multiple trap levels of different origins, such as vacancies, antisite defects, interstitial atoms, and grain-boundary-derived defects, are present. These defects create both shallow and deep energy levels within the band gap, complicating carrier transport and recombination mechanisms. In particular, non-homogeneous defect distributions can cause local potential fluctuations in intragrain and grain boundary regions, leading to spatial variation of the electric field and disordered carrier transport. Their defect impact on device performance is usually described *via* the SRH recombination model. SRH recombination occurs when electrons and holes recombine through localized defect states within the band gap of a semiconductor.^34^ SRH recombination involves electron–hole pairs recombining through trap states located within the semiconductor's energy gap. Typically centered at mid-gap, an electron from the conduction band occupies the defect energy level (*E*_t_), where it recombines with a valence-band hole, releasing energy as a photon. The solar cell's short-circuit current density (*J*_sc_) is adversely affected by increasing neutral defect concentrations (*N*_t_), as the carrier lifetime shortens. The corresponding SRH trap-assisted recombination process is described in eqn (8):8
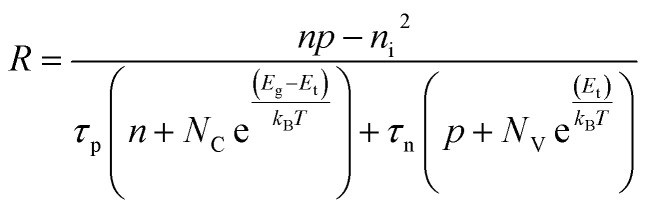
*p* and *n* indicate the densities of free holes and electrons within a semiconductor, while *E*_t_ denotes the energy levels of trap-related defects. The defect density specified in eqn (9) acts as a fundamental parameter controlling the recombination rate (*R*).^35^ Electron and hole effective lifetimes, represented by *τ*_n_ and *τ*_p_, respectively, can be determined through the relation given in eqn (9).^36^9
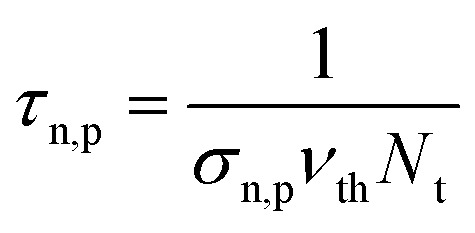
10
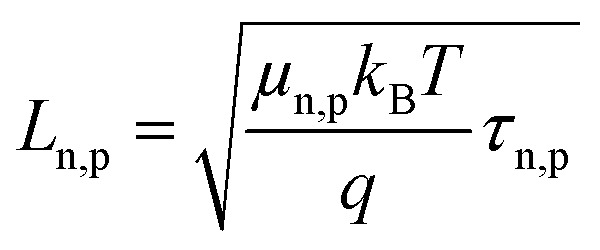


Capture cross-sections for electrons and holes are expressed as *σ*_n,p_, while *N*_t_ and *ν*_th_ correspond to trap density and carrier thermal velocity. The relationship between carrier lifetime and diffusion length is provided in eqn (10). *µ*_e_ and *µ*_p_ describe the carrier mobilities of electrons and holes, respectively, and *q* represents the elementary charge inherent to these carriers. These expressions suggest that higher defect concentrations in the semiconductor act as efficient recombination centers, resulting in shorter carrier lifetimes and a subsequent decline in solar cell efficiency.^35,37,38^ No significant changes in solar cell performance were detected for *N*_t_ values spanning from 1 × 10^12^ cm^−3^ to 5 × 10^15^ cm^−3^, suggesting a stable operational regime. Since the carrier lifetime and diffusion length remained sufficiently high, no significant change in device performance was observed. Electrons and holes formed in this region can reach the junction region without significant trapping or recombination and can be effectively collected. A substantial increase in defect density from 1 × 10^16^ cm^−3^ to 5 × 10^20^ cm^−3^^39^ results in a marked reduction in device performance, with *V*_oc_, *J*_sc_, FF, and efficiency decreasing significantly to 0.34 V, 7.99 mA cm^−2^, 32.30%, and 1.75%, respectively, as depicted in Fig. 4(e–h). The increase in *N*_t_ increased the density of trap-assisted recombination centers within the absorber layer and caused a significant decrease in electron and hole lifetimes, as shown in eqn (9). Consequently, the carrier diffusion length shortened, photo-excited carriers had difficulty reaching the junction region, and recombination losses increased significantly.

**Fig. 5:**
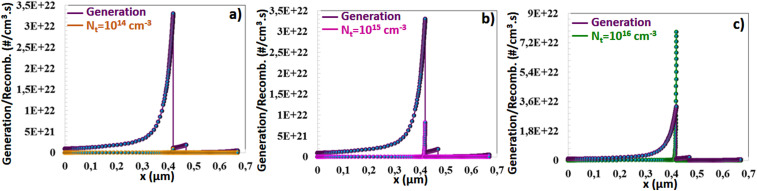
The position-dependent generation and recombination characteristics of the CIGSe solar cell for defect densities (*N*_t_) ranging from 10^14^ (a), 10^15^ (b), and 10^16^ cm^−3^ (c).

A high level of photo-induced carrier generation is localized near the depletion zone of p-type absorber, with values approaching 3.3 × 10^22^ (cm^−3^ s^−1^) at *x* = 0.42 µm, as illustrated in Fig. 5(a). Due to the declining absorption of incident photons toward the back contact, the photogeneration rate is reduced to approximately 9.12 × 10^20^ (cm^−3^ s^−1^) at *x* = 0 µm. As depicted in Fig. 5(b), the generation–recombination profile across the active layer indicates that within the defect density interval of 10^14^ cm^−3^ to 10^16^ cm^−3^, SRH recombination does not exceed the generation rate until *N*_t_ reaches to 10^15^ cm^−3^. The results indicate that above a critical defect density of 10^15^ cm^−3^, SRH recombination increases significantly, negatively impacting PV efficiency, as seen in Fig. 4(e–h).

#### 2.4.2. The impact of the radiative recombination

In CIGSe solar cells, the radiative recombination mechanism is not limited to ideal band-to-band transitions; it is also influenced by multilevel defect states, band-tail formation, and spatially inhomogeneous carrier distributions. Specifically, grain boundaries, compositional fluctuations, and local potential variations can locally increase or suppress the probability of radiative transitions by altering the spatial overlap of electrons and holes. Therefore, the radiative recombination coefficient (*B*_r_) exhibits complex behavior related to defect distribution and local electronic structure, rather than being a uniform and constant parameter in real devices. A photon is emitted when an electron recombines with a hole by transitioning from the conduction band to the valence band, a process termed radiative recombination.^40^ The efficiency and rate of this mechanism are captured by the radiative recombination coefficient of *B*_r_ as described in eqn (11):11
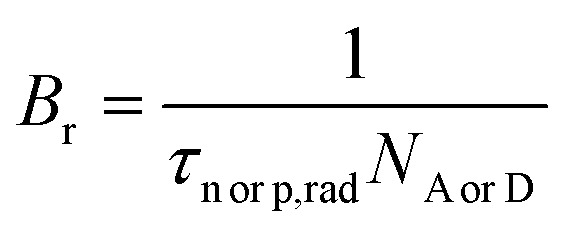


**Fig. 6:**
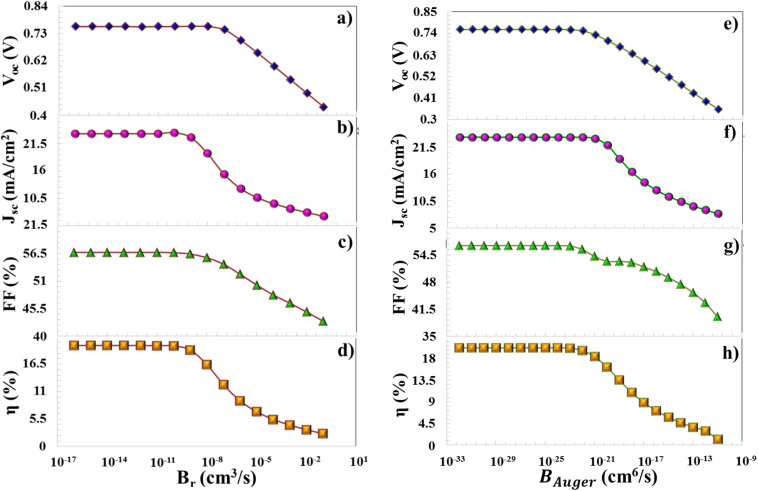
Variation of PV parameters: *V*_oc_, *J*_sc_, FF, *η* as a function of (a–d) *B*_r_ (cm^3^ s^−1^) and (e–h) AECC (cm^6^ s^−1^).

The radiative lifetimes of carriers, where electrons and holes are described by *τ*_n,rad_(s) and *τ*_p,rad_(s), respectively.^40^ The performance of CIGSe solar cell, in terms of efficiency, was found to be stable and unaffected by changes in *B*_r_ over the investigated range (from 10^−16^ cm^3^ s^−1^ to 10^−8^ cm^3^ s^−1^), which shows that the rate of radiative recombination is quite low compared to the carrier production rate. Therefore, photo-generated electron–hole pairs dissociate efficiently, and carrier collection losses are minimal. With increasing *B*_r_ over the range of 10^−8^ cm^3^ s^−1^ to 10^−1^ cm^3^ s^−1^,^21,41^ the device exhibited a substantial decline in the PV parameters, including a notable drop in *V*_oc_ (0.434 V), *J*_sc_ (6.93 mA cm^−2^), FF (42.98%), and conversion efficiency (2.58%) as seen in Fig. 6(a–d). The enhancement of *B*_r_ is associated with shortened carrier lifetimes or reduced carrier concentrations, ultimately leading to a decline in device performance, as shown in eqn (11). Increased radiative recombination reduces the carrier lifetime, causing photo-generated carriers to recombine before reaching the electrodes. In particular, according to eqn (11), the increase in *B*_r_ directly corresponds to the reduction in carrier lifetimes. This reduction in carrier lifetime leads to a *V*_oc_ loss by narrowing the half-Fermi-level separation, while also limiting *J*_sc_ and FF by reducing the effective carrier density. The recombination rate approaching and even exceeding the production rate in some regions limited net carrier production. This situation particularly weakened the effective charge separation in the absorber layer and reduced the carrier collection efficiency. As a result, the device efficiency dropped to 2.58%.

**Fig. 7:**
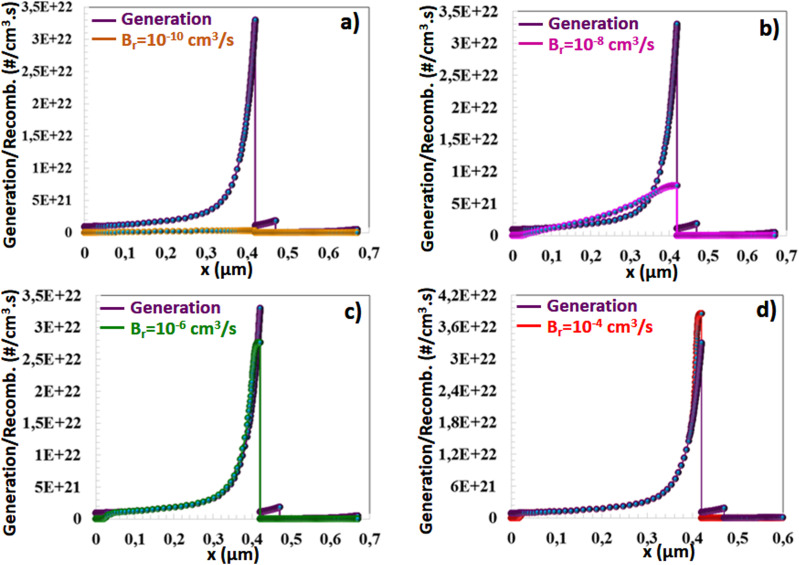
The position-dependent generation and recombination characteristics of the CIGSe solar cell for the radiative recombination coefficient of (*B*_r_) ranging from 10^−10^ (a), 10^−8^ (b), 10^−6^ (c), 10^−4^ cm^−3^ (d).

According to the recombination-position graph shown in Fig. 7, the recombination rate is 3.22 × 10^20^, 7.76 × 10^21^, 2.77 × 10^22^, 3.85 × 10^22^ (cm^−3^ s^−1^) for *B*_r_ = 10^−10^, 10^−8^, 10^−6^, 10^−4^ cm^3^ s^−1^ at *x* = 0.420 µm, respectively. For *B*_r_ = 10^−10^ and 10^−8^ cm^3^ s^−1^, since the recombination rates are significantly lower than the generation rates, the photovoltaic parameters do not change in this range as demonstrated in Fig. 6(a–d). However, for the range of *B*_r_ = 10^−6^ and 10^−4^ cm^3^ s^−1^, since the recombination rate is close to and sometimes exceeds the generation rate, charge generation is limited, and photovoltaic performance begins to degrade.

#### 2.4.3. The effect of the Auger electron recombination

The recombination of charge carriers within the absorber semiconductor leads to energy release, which is internally transferred to other carriers, facilitating non-radiative excitation to higher energy states, a phenomenon referred to as Auger recombination. Eqn (12) provides the formal definition of the Auger recombination coefficient.^42^12
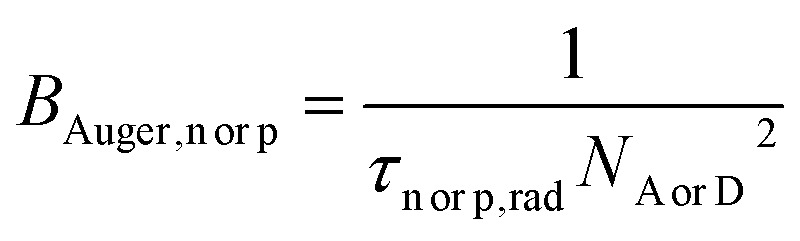


**Fig. 8:**
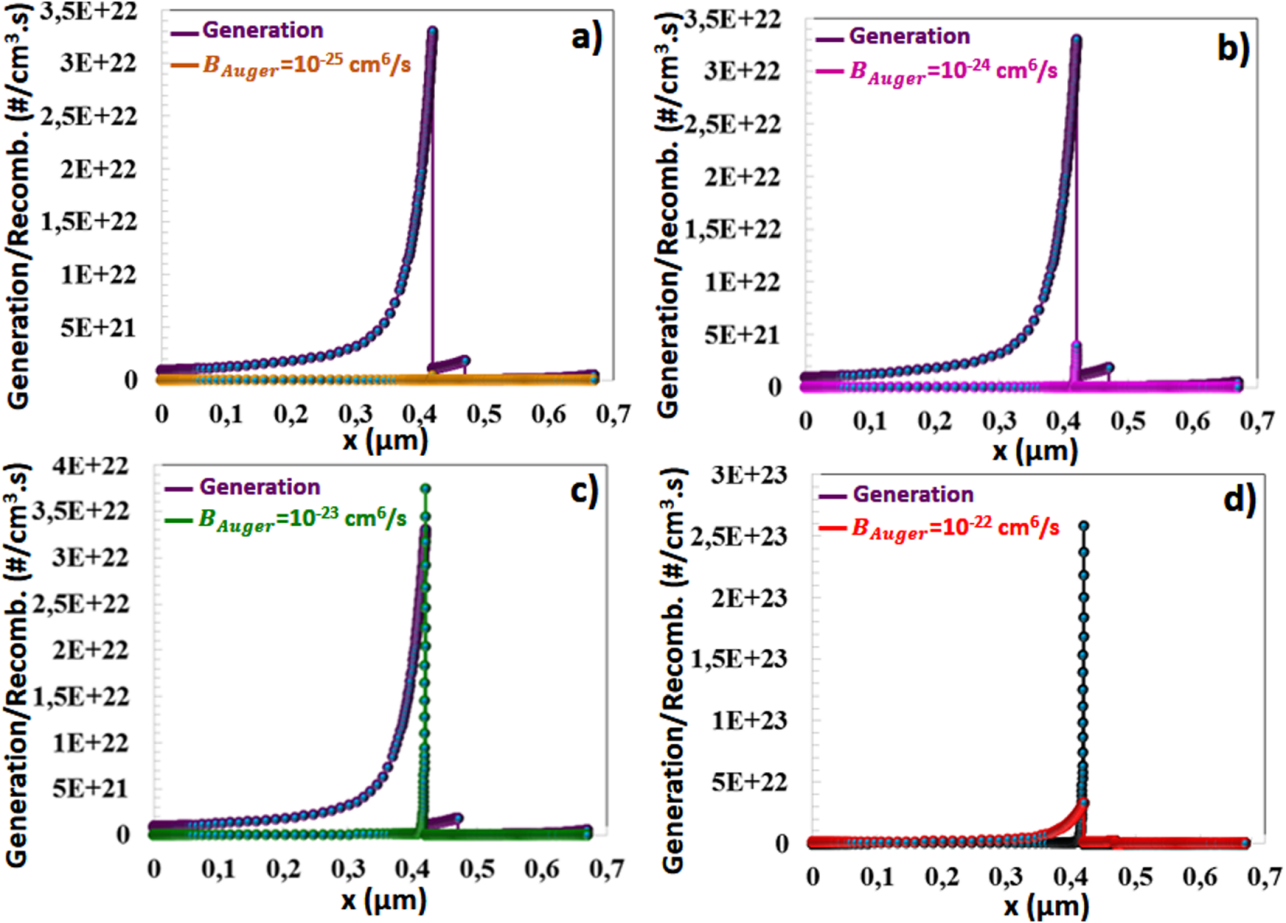
The position-dependent generation and recombination characteristics of the CIGSe solar cell for the Auger electron recombination coefficients (*B*_Auger_) of 10^−25^ (a), 10^−24^ (b), 10^−23^ (c), 10^−22^ cm^−3^ (d).

This work assessed the solar cell's PV parameters as functions of the Auger recombination coefficient, spanning 10^−32^ cm^6^ s^−1^ to 10^−23^ cm^6^ s^−1^, and observed notable deviations in all PV parameters for Auger recombination coefficients up to 10^−22^ cm^6^ s^−1^. As shown in the graph in Fig. 8, in the range 10^−25^ cm^6^ s^−1^ to 10^−24^ cm^6^ s^−1^,^43–45^ the generation rate exceeds the recombination rate, and this does not affect the PV parameters. So, the majority of photocarriers produced in the system reach the junction region without undergoing recombination. Therefore, the production rate remained dominant over the recombination rate, and no significant change in PV values occurred. However, at the value of 10^−22^ cm^6^ s^−1^, since the amount of recombination (2.58 × 10^23^ (cm^−3^ s^−1^)) significantly exceeds the generation rate (3.3 × 10^22^ (cm^−3^ s^−1^)), a decrease in PV parameters begins, and the *V*_oc_, *J*_sc_, FF and efficiency parameters for 10^−11^ cm^6^ s^−1^ drops to 0.352 V, 7.94 mA cm^−2^, 39.68% and 1.22%, respectively. The recombination rate exceeding the production rate led to a significant portion of the carriers formed in the absorber layer being lost before reaching the contacts. This results in a significant reduction in carrier lifetime and a narrowing of the separation between the quasi-Fermi levels. The reduction in quasi-Fermi level separation directly decreases *V*_oc_, while the decrease in carrier collection efficiency due to the short diffusion length reduces *J*_sc_.^43–45^ At the same time, increased internal losses and impaired carrier transport also reduce the FF. Multi-level trap structures and non-homogeneous defect distributions can complicate the effects of Auger recombination. Deep-level defects lead to carrier localization, while carrier–carrier interactions increase in regions with high carrier density, accelerating Auger processes. Furthermore, local potential barriers formed at grain boundaries can restrict carrier transport, leading to additional losses in *J*_sc_ and FF parameters.^43–45^ Although the idealized approach used in the SCAPS model can explain the general trend, spatial heterogeneity and defect-induced local carrier accumulations in real devices can make Auger recombination more pronounced.

### 2.5. The impact of series and the shunt resistance

The series resistance (*R*_s_) and shunt resistance (*R*_sh_) are the dominant parasitic resistances in solar cells. (*R*_s_) arises from the charge carriers' transport across the semiconductor–metal interface and the cumulative internal resistances within the device.^46^ Moreover, the series resistance is governed by multiple factors, including surface grain boundaries, lattice strain, interface trap states, and the intrinsic resistance of the thin-film surface. Shunt resistance fundamentally originates from structural imperfections introduced during thin-film production, including microcracks and pinhole defects,^46^ as well as parasitic current pathways and junction-associated leakage mechanisms that collectively degrade device integrity.

**Fig. 9:**
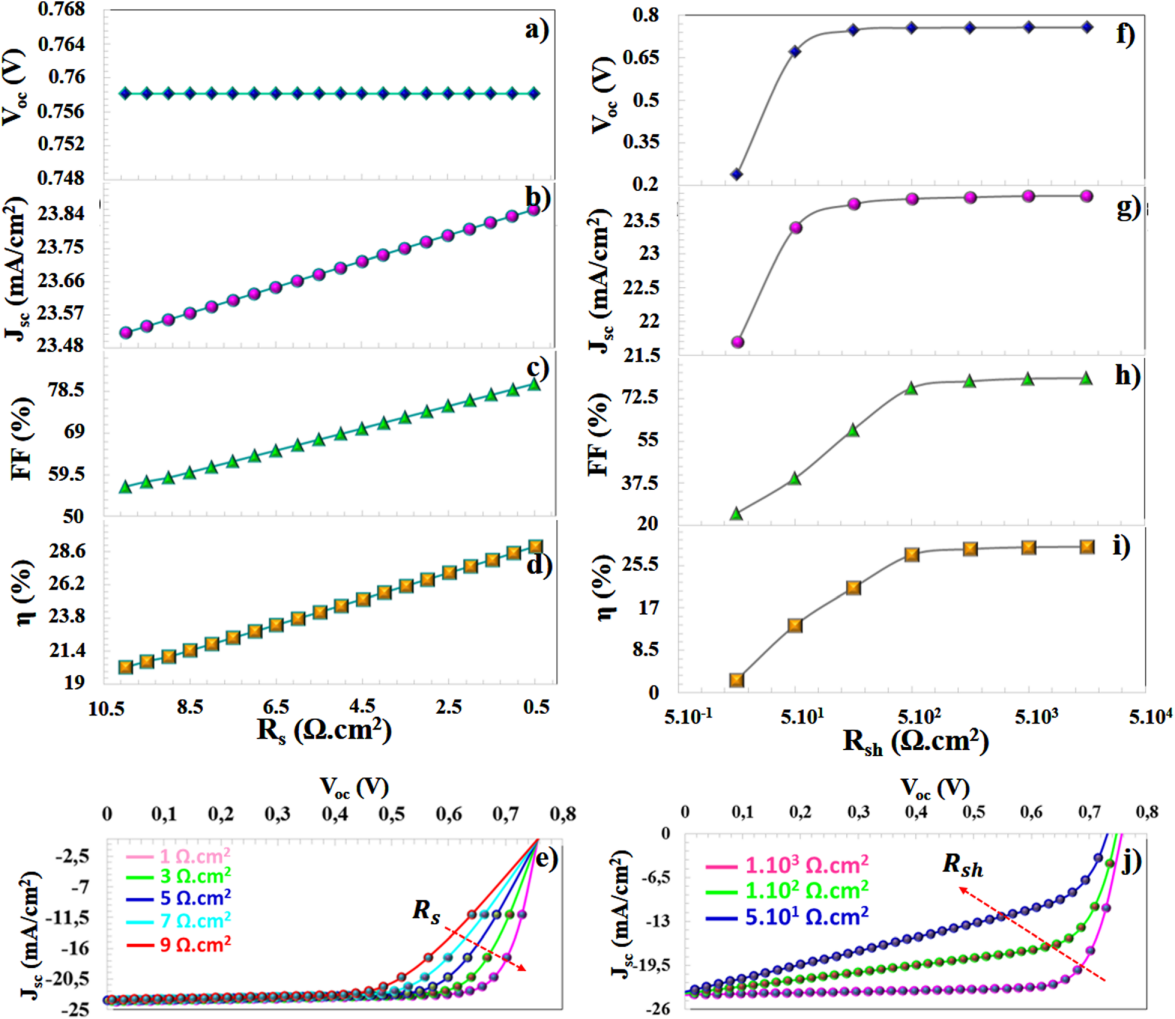
Variation of PV parameters: *V*_oc_, *J*_sc_, FF, *η* as a function of (a–d) *R*_s_ (Ω cm^2^) and (e–h) *R*_sh_ (Ω cm^2^).

The role of *R*_s_ and *R*_sh_ in modulating the fundamental PV characteristics is explicitly captured through the formulation presented in eqn (13):13
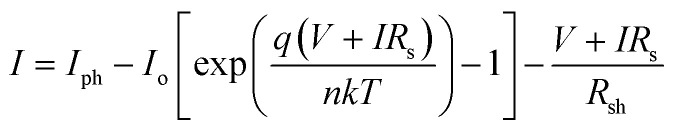
where, *I* corresponds to the output current,*I*_o_ denotes the saturation current, *I*_ph_ represents the photocurrent, and *V* signifies the applied voltage, while *q*, *k*, *n*, and *T* refer to the elementary charge, Boltzmann constant, ideality factor, and absolute temperature, respectively. *R*_s_ was systematically reduced from 10 Ω cm^2^ to 1 Ω cm^2^, while *R*_sh_ was concurrently varied over a broad range from 1 × 10^1^ Ω cm^2^ to 1 × 10^4^ Ω cm^2^. Although *V*_oc_ remains largely unaffected by the decrease in series resistance, a significant increase in *J*_sc_, FF, and conversion efficiency is observed, as shown in Fig. 9(a–e). A reduction in ohmic losses, coupled with more efficient charge carrier collection and enhanced current extraction to the external circuit, results in significant improvements in *J*_sc_, FF, and overall efficiency. A decrease in *R*_s_ from 10 Ω cm^2^ to 1 Ω cm^2^ led to pronounced increases in *J*_sc_, FF, and power conversion efficiency, with values rising from 23.52 to 23.83 mA cm^−2^, 56.77% to 78.98%, and 20.25% to 28.52%, respectively.^21,47–51^ High *R*_s_ can disrupt the local electric field distribution within the device. When carriers have difficulty reaching the contacts, accumulation occurs within the absorber, increasing the probability of recombination. Carriers accumulating, especially at grain boundaries, can recombine at trap levels. When the *R*_s_ value decreases, ohmic losses are significantly reduced, charge carrier accumulation is prevented, and maximum power transfer is improved. Furthermore, the contacts achieve high conductivity, a low-defect crystal structure, a large grain size, reduced grain boundary density, good band alignment, and high-quality metal-back-contact/semiconductor interfaces.

Elevating the shunt resistance reduces current leakage across the shunt path, thereby improving *V*_oc_ and *J*_sc_. This increase also results in notable enhancements of *V*_oc_, *J*_sc_, FF, and *η*, as illustrated in Fig. 9(f–l). For the limiting case of an infinitely large shunt resistance, the output voltage can be expressed as follows:14
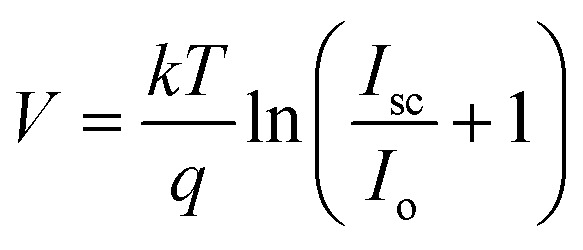


As indicated in eqn (14), the output voltage is inversely affected by the magnitude of the reverse saturation current (*I*_0_). A leakage current within the junction can be attributed to sagging bonds, defects, cracks, and pinholes introduced during thin-film deposition. A decrease in shunt resistance directly contributed to the deterioration of all PV device characteristics, as shown in Fig. 9(f–i). Increasing *R*_sh_ limited parallel leakage current paths within the cell, directing the photogenerated current to the external circuit. This improved *V*_oc_ and *J*_sc_ values, particularly in the low-voltage region, by reducing carrier recombination *via* alternating leakage paths. An increase in *R*_sh_ indicates suppression of ohmic leakage through interface defects, pinhole formation, grain-boundary conductivity, and deposition-induced microcracks. Reducing these leakage paths creates a device characteristic closer to ideal diode behavior and facilitates redirecting photogenerated current toward charge collection rather than recombination.

### 2.6. The effect of different BSF layers

**Fig. 10:**
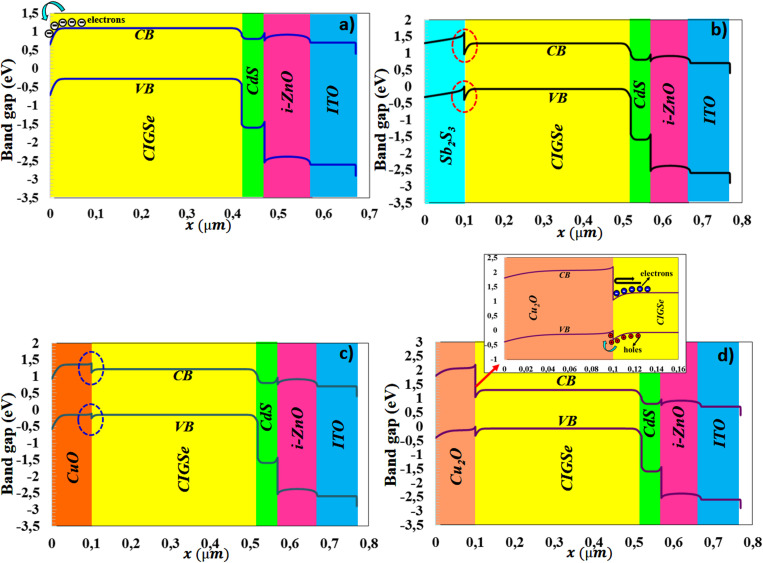
Energy band diagrams of a Au/CIGSe/CdS/i-ZnO/ITO solar cell illustrating (a) the absence of a BSF layer, and the incorporation of BSF layers composed of (b) Sb_2_S_3_, (c) CuO, and (d) Cu_2_O.

The BSF in solar cells is formed by a highly doped p-type (p^+^) layer at the interface between the rear contact and the light-absorbing layer, acting as a barrier to minimize carrier recombination and enhancing overall device efficiency.^6^ It facilitates the directed transport of electrons, minimizing recombination losses and thereby enabling the device to achieve its maximum PV efficiency. Electrons reaching the rear interface of CIGSe solar cells are commonly trapped, limiting their ability to contribute to the generated current (in Fig. 10(a)). The BSF layer facilitates more efficient electron transport by functioning as a quasi-ohmic contact, establishing a modest built-in potential that directs the electrons back, as illustrated in the energy band diagram in Fig. 10(b–d). The BSF layer directs electrons from the conduction band of CIGSe back toward the depletion region, facilitating their effective collection.^11,52^ In this manner, even minority carriers are effectively collected at the heterojunction interface rather than lost to recombination at the rear contact, thereby enhancing *J*_sc_.

CIGSe-based ultra-thin film solar cells with absorber layers reduced to 420 nm could face limitations in achieving elevated open-circuit voltages. The inherently fragile layers of ultra-thin PV devices mean that even minimal electron losses can significantly impact energy conversion. The implementation of a BSF layer curtails recombination of minority carriers at the rear electrode, enhancing *V*_oc_ and supporting more efficient collection of photogenerated charges.^53^ Optimal band alignment between the BSF and CIGSe layers facilitates smooth hole transport from the absorber to the BSF, reducing carrier losses and improving both *V*_oc_ and *J*_sc_.^11^ With well-matched energy levels between the CIGSe and BSF layers, hole transport to the rear electrode becomes more efficient, resulting in minimal losses and reduced recombination, thereby supporting enhanced charge collection and optimal device operation.

**Fig. 11:**
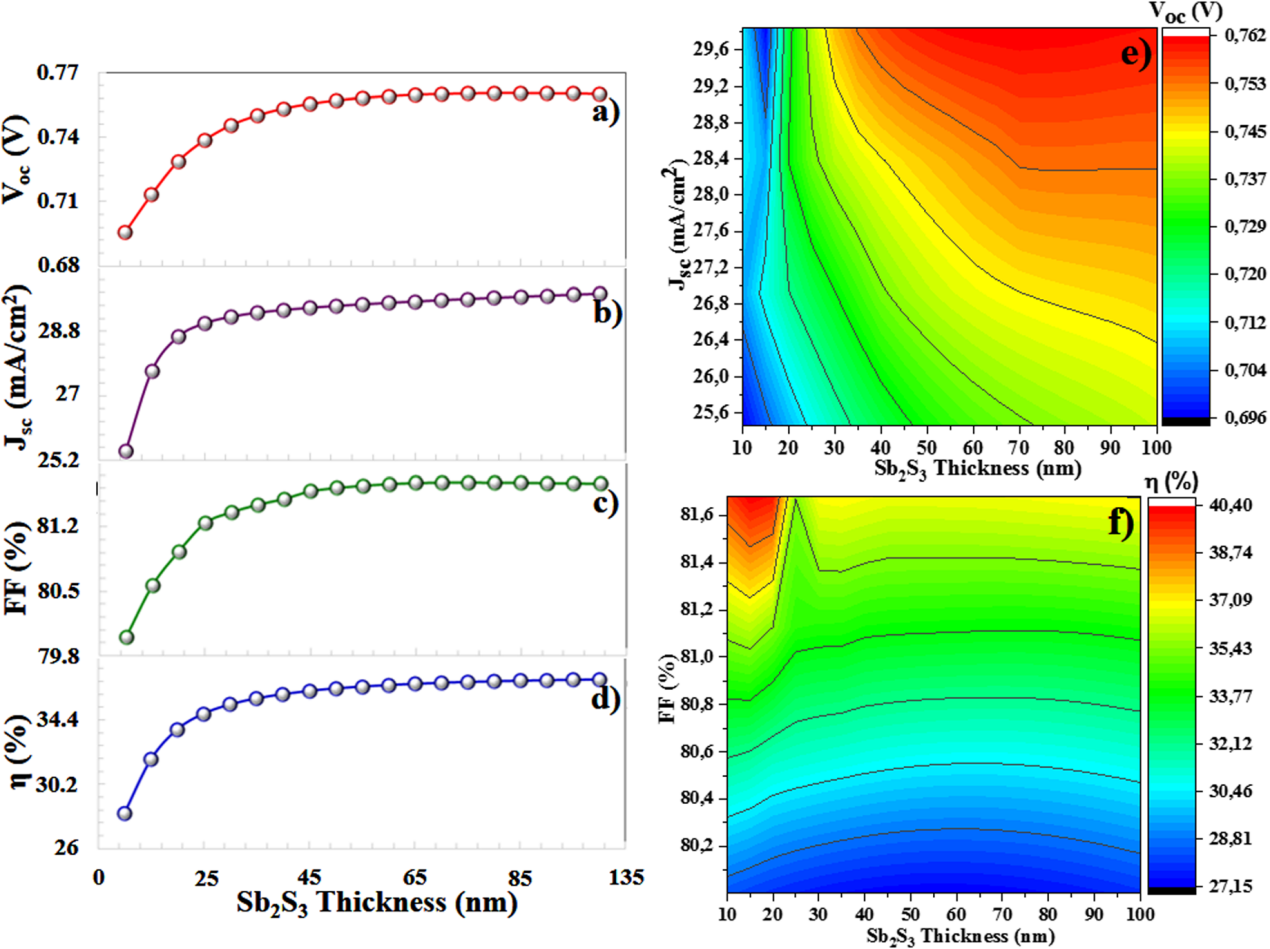
(a–d) The variation of *V*_oc_, *J*_sc_, FF, and *η*, and (e and f) the corresponding contour plots with respect to the thickness of the Sb_2_S_3_ BSF layer.

As observed in Fig. 11(a), the absence of a BSF layer in the CIGSe solar cell eliminates the back-contact barrier, allowing minority carriers to travel freely to the metal electrode.^4^ This facilitates interfacial recombination, thereby reducing device efficiency. In this work, BSF layers comprising Sb_2_S_3_, CuO, and CuO_2_ were incorporated to boost device efficiency, and their corresponding physical parameters are summarized in Table 1. Fig. 10(d) illustrates that the Cu_2_O-based BSF layer forms an energy barrier that suppresses minority-carrier flow to the rear contact, redirecting them into the active layer. In contrast, valence-band holes can reach the back contact with negligible recombination losses.

Increasing the BSF thickness enhances the electrostatic field and stabilizes the potential barrier at the p^+^-BSF/p-type CIGSe absorber interface, thereby reducing carrier leakage and SRH recombination near the back contact. A thicker BSF also improves back-surface passivation and strengthens carrier separation, which is particularly beneficial for ultra-thin CIGSe absorbers where carriers can easily reach the rear contact. Consequently, carrier collection efficiency increases while back-surface recombination losses decrease. When the Sb_2_S_3_ BSF layer thickness rose from 10 nm to 100 nm, notable improvements in device performance were observed: *V*_oc_, *J*_sc_, FF, and *η* values, which enhanced from 0.695 V to 0.760 V, from 25.45 mA cm^−2^ to 29.84 mA cm^−2^, from 80.00% to 81.66%, and from 28.33 to 37.05%, respectively (Fig. 11(a–d)). The band gap of 1.62 eV for Sb_2_S_3_ forms a back face with a wider band than the absorber. The low electron affinity (3.7 eV) creates a repulsive potential barrier for electrons at the CIGSe interface. This reduces recombination by pushing electrons that would otherwise move towards the back contact back into the absorber. The efficiency for an Sb_2_S_3_ film thickness of 15 nm is approximately 31%, while the yield for a thickness of 20 nm is approximately 33%. The Shockley–Queisser (SQ) limit for a single-junction CIGS solar cell is approximately 30% to 33.7% under standard AM1.5G solar spectrum conditions. Accordingly, the most realistic value was obtained for 10 nm Sb_2_S_3_.

**Fig. 12:**
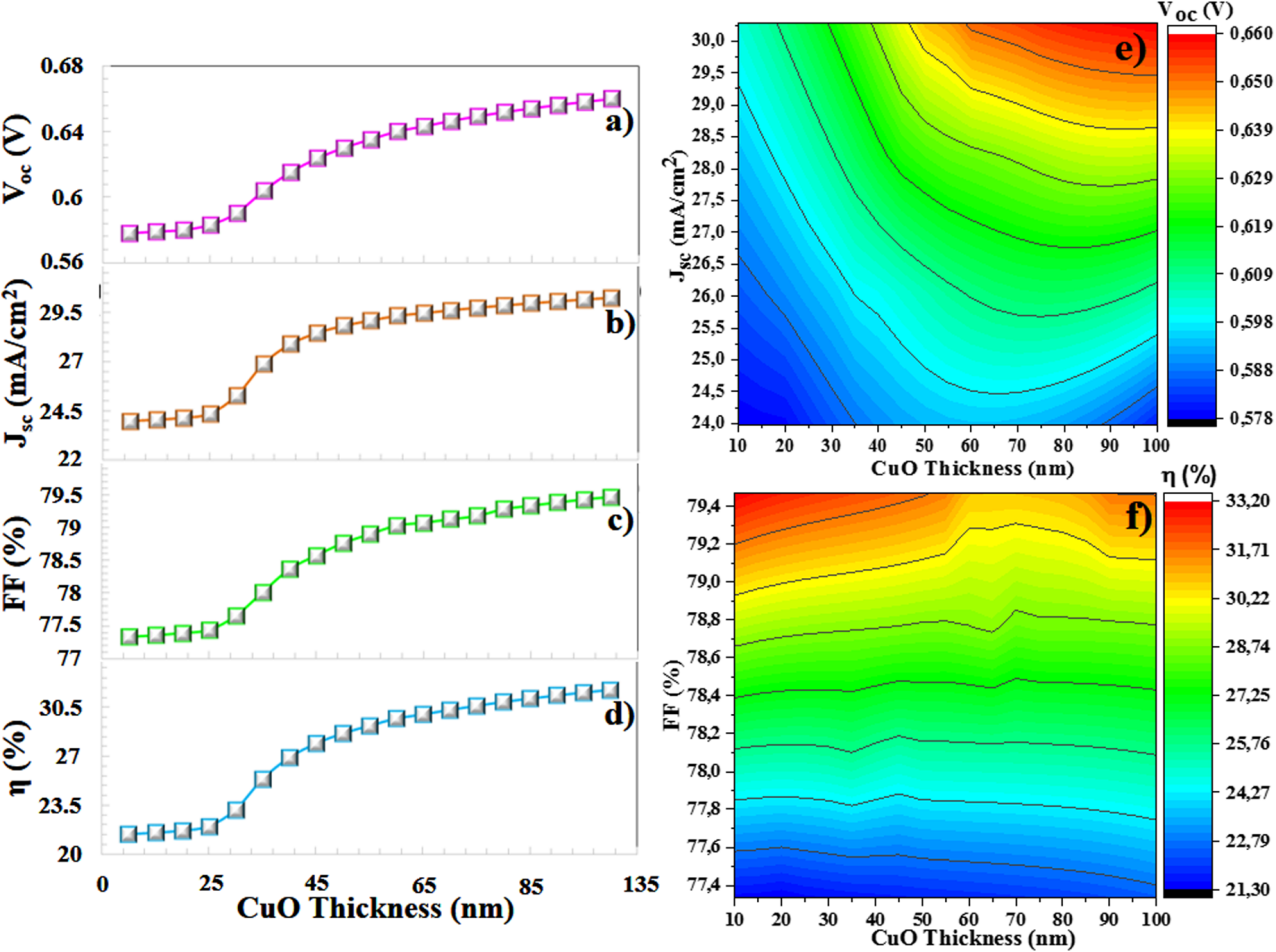
(a–d) The variation of *V*_oc_, *J*_sc_, FF, and *η*, and (e and f) the corresponding contour plots with respect to the thickness of the CuO BSF layer.

*J*_sc_, *V*_oc_, FF and power conversion efficiency parameters of modelled CIGSe solar cell, based on a heavily doped p-type CuO semiconductor with a 1.51 eV band gap, exhibit an increase from 23.98 mA cm^−2^ to 30.27 mA cm^−2^, from 0.578 V to 0.660 V, from 77.33% to 79.47%, from 21.44% to 31.76% in Fig. 12(a–d), with increasing CuO BSF thickness from 10 nm 100 nm, respectively. Because the band gap of CuO is lower than that of Sb_2_S_3_ (1.62 eV), the voltage built-in and barrier height are lower, and some of the incident minority charge carriers may undergo recombination in the back contact region, resulting in somewhat lower performance than that of a solar cell with a Sb_2_S_3_ BSF layer. CuO exhibits high p-type conductivity, a deep valence band, and a high hole density, facilitating hole transport to the back contact. In addition, CuO has a higher electron affinity (4.07 eV), which reduces the conduction band discontinuity with CIGSe, and electron reflection may not be as strong as in Sb_2_S_3_. CuO has relatively low hole mobility, which can limit carrier transport and increase series resistance, especially when the BSF thickness increases. Therefore, the solar cell with a CuO BSF exhibits lower PV performance than those with other BSF layers.

**Fig. 13:**
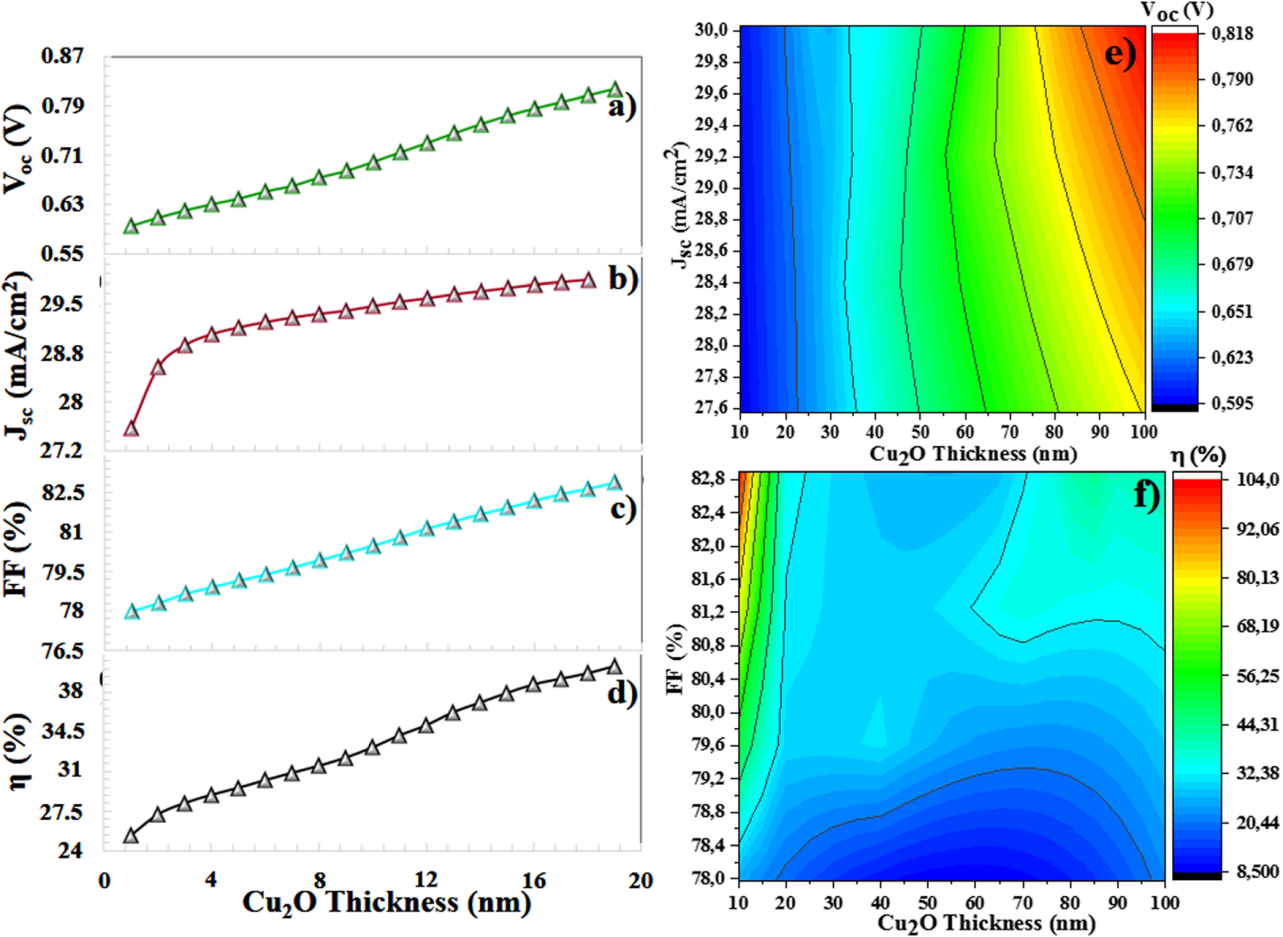
(a–d) The variation of *V*_oc_, *J*_sc_, FF, and *η*, and (e and f) the corresponding contour plots with respect to the thickness of the Cu_2_O BSF layer.

Cu_2_O exhibits high-doping p^+^-type semiconductor behavior with a wide band gap (2.2 eV) and high hole mobility. Enhancing the Cu_2_O BSF layer thickness from 10 nm to 100 nm significantly improves the performance of the CIGSe solar cell device, yielding increases in *J*_sc_, *V*_oc_, FF, and power conversion efficiency from 27.57 to 30.03 mA cm^−2^, 0.595 V to 0.817 V, 77.98% to 82.88%, and 25.35% to 40.34% in Fig. 13(a–d), respectively. The band gap of the Cu_2_O layer (2.2 eV) is larger than that of other BSF layers. Therefore, the higher barrier and built-in voltage formed against electrons (photo-excited minority charge carriers) in the active layer generate a larger electrical field, pushing the majority of electrons back towards the depletion region and increasing the amount of charge collection. Cu_2_O has the widest band gap (2.2 eV), forming a strong energy barrier in the back region and effectively preventing electrons from reaching the back contact. Because Cu_2_O has a wide band gap, it can exhibit low absorption across much of the visible region. Thus, it can reduce parasitic absorption, help long wavelengths remain within the absorber, and increase the optical path length when used with back-reflector structures. Its low electron affinity (3.2 eV) forms a significant conduction band offset with CIGSe (Δ*E*_C_ = *χ*_CIGSe_ − *χ*_Cu_2_O_). This structure reflects minority-carrier electrons back into the absorber, drastically reducing back-surface recombination and providing a strong improvement, especially in *V*_oc_. Furthermore, Cu_2_O has very high hole mobility, which greatly facilitates hole transport to the back contact and minimizes series resistance losses. High electron mobility also supports carrier transport. Due to the high acceptor density, a strong p^+^ field is formed,^54,55^ and strong band bending occurs in the back region. Therefore, Cu_2_O can exhibit the most effective BSF behavior in terms of both electrostatic field and energy barrier.

The high-efficiency value of CIGSe with Cu_2_O BSF, a theoretical result obtained from SCAPS-1D simulation, is not an experimentally verified device performance. The BSF layer acts as a reflective barrier, particularly for minority-carrier electrons, significantly reducing recombination at the back contact. Thus, back surface recombination losses are suppressed, carrier collection efficiency is increased, band alignment becomes more favorable, recombination due to interface defects is reduced, and carrier lifetime and diffusion length are improved. A 35 nm BSF layer provides a partial barrier at the back surface, reducing recombination to a limited extent, and the efficiency is approximately 30%. When the BSF thickness is increased to 55 nm, the band curve and potential barrier become more pronounced, allowing more effective reflection of minority carrier electrons at the back contact. As a result, carrier collection efficiency increases to 35%. At a BSF thickness of 100 nm, back surface recombination is almost completely suppressed, and carrier losses are minimized under ideal simulation conditions. This situation leads to simultaneous increases in *J*_sc_, *V*_oc_, and the charge factor, bringing the efficiency to approximately 40%. According to the Shockley–Queisser limit, the most realistic value was obtained for Cu_2_O thicknesses below 35 nm.

### 2.7. The effect of interface defect (*N*_t_) between CIGSe and BFS layers on the efficiency of the solar cell

**Fig. 14:**
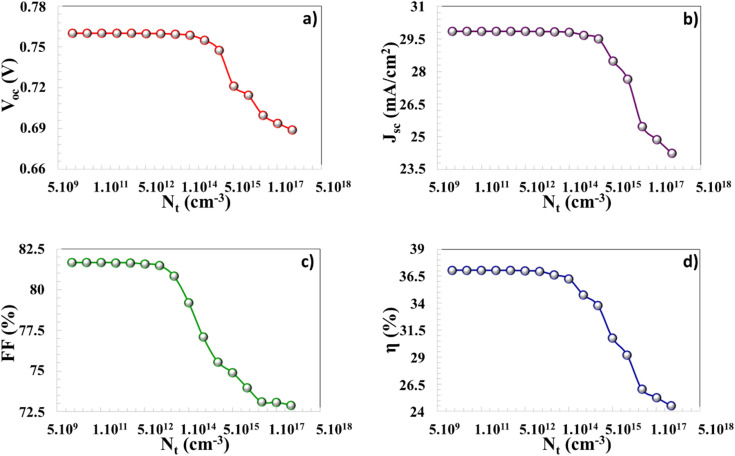
(a–d) Variation of PV parameters of the CIGSe solar cell with Sb_2_S_3_ BSF layer: *V*_oc_, *J*_sc_, FF, *η* as a function of *N*_t_ (cm^−3^).

The lattice mismatch between CIGS in a tetragonal phase, Sb_2_S_3_, CuO, and Cu_2_O BSF layers can lead to strain, dislocations, and recombination centers in experimentally produced thin-film solar cells. Increasing the thickness of the BSF may increase the distance carriers travel, leading to carrier scattering, point defects (vacancies, interstitials), grain boundaries, dopant irregularities, composition fluctuations, increased chemical reactions and interdiffusion, decreased conductivity, and, consequently, an increase in *R*_s_. Excessively thick or non-uniform BSF layers may cause pinholes, microcracks, leakage currents, and local recombination losses in real thin-film solar cells. Fig. 14 shows the effect of interface defect density (*N*_t_) (ranging from 1 × 10^10^ cm^−3^ to 5 × 10^17^ cm^−3^) between the Sb_2_S_3_ and CIGS layers on PV parameters. There is no significant change in PV parameters for *N*_t_ = 1 × 10^10^ cm^−3^ to 1 × 10^13^ cm^−3^. However, when *N*_t_ increases from 5 × 10^13^ cm^−3^ to 5 × 10^17^ cm^−3^, *V*_oc_, *J*_sc_, FF, and *η* values drop from 0.760 V to 0.689 V, from 29.84 mA cm^−2^ to 24.24 mA cm^−2^, from 81.65% to 72.91%, from 37.05% to 24.52%, respectively.

**Fig. 15:**
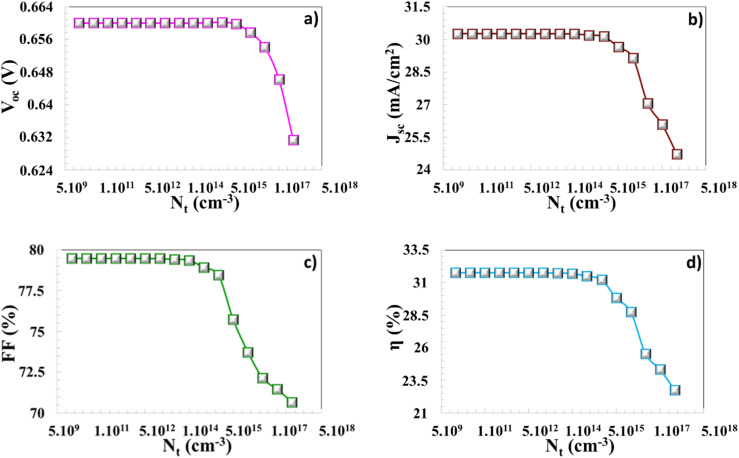
(a–d) Variation of PV parameters of the CIGSe solar cell with CuO BSF layer: *V*_oc_, *J*_sc_, FF, *η* as a function of *N*_t_ (cm^−3^).

**Fig. 16:**
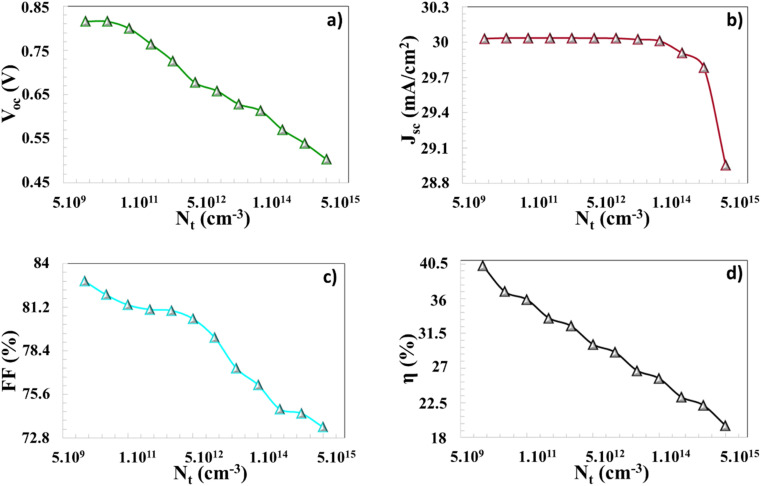
(a–d) Variation of PV parameters of the CIGSe solar cell with Cu_2_O BSF layer: *V*_oc_, *J*_sc_, FF, *η* as a function of *N*_t_ (cm^−3^).

According to Fig. 15, no significant change was observed in the PV parameters of the CIGSe solar cell with CuO BSF layer for *N*_t_ = 1 × 10^10^ cm^−3^ to 1 × 10^14^ cm^−3^. When *N*_t_ rose from 1.1014 cm^−3^ to 5.1017 cm^−3^, *V*_oc_, *J*_sc_, FF, and *η* values decreased from 0.660 V to 0.631 V, from 30.273 mA cm^−2^ to 24.72 mA cm^−2^, from 79.47 to 70.67%, from 31.76% to 22.77%, respectively. For Sb_2_S_3_ and CuO BSF layered interface defects, solar cells exhibited similar PV performance. The effect of *N*_t_ on CIGSe solar cell with Cu_2_O BSF layer is presented in Fig. 16. There is no significant change in *J*_sc_ values until *N*_t_ = 1 × 10^14^ cm^−3^. While *V*_oc_, *J*_sc_, FF and *η* are 0.815 V, 3030 mA cm^−2^, 82.88%, 40.34% for *N*_t_ = 1 × 10^10^ cm^−3^, *V*_oc_, *J*_sc_, FF and *η* are 0.503 V, 28.95 mA cm^−2^, 73.49%, 19.58% for *N*_t_ = 5 × 10^15^ cm^−3^, respectively. The Cu_2_O BSF solar cell was more affected by the *N*_t_ value compared to the others (Table 2).

**Table 2:** Comparison of PV parameters of CIGSe thin-film solar cells modeled with the SCAPS-1D program reported in the literature

Solar cell (experimental: E, theoritacal: T, back surfac field: BSF)	*V*_oc_ (V)	*J*_sc_ (mA cm^−2^)	FF (%)	*η* (%)	Reference
Ag/ZnO:Al/i-ZnO/CdS/CIGS/Mo (T)	0.733	36.35	83.33	22.23	56
ZnO:Al/ZnS/CIGSe/Mo (T)	0.693	38.06	83.71	22.11	57
ZTO/CIGS (T)	0.89	36.0	82.0	31.10	58
ITO/CIGS/GaSe (T)	1.62	24.03	94.5	25	59
Zn(O,S)/CIGS/Si/Mo (T)	0.74	39.72	81.94	24.16	60
Mo/CIGS/CdSe/ZnO/ITO (T)	0.983	33.63	75.93	25.11	61
i-ZnO/n-CdS/p-CIGS/Mo (T)	0.80	38.20	75.00	26.15	62
FTO/SnS_2_/CIGS/Sb_2_S_3_/Ni (T)	1.08	33.75	88.50	31.15	63
n-ZnO: Al/i-ZnO/n-ZnS/p-CIGS/Mo (T)	0.82	36.90	85.50	26.00	64
ZnO/CdS/CIGS/Mo (T)	0.78	38.66	80.00	24.45	65
ZnO:Al/i-ZnO/CdS/CIGS/Mo (T)	0.76	32.04	71.47	17.49	66
ZnO:Al/ZnMgOCdS/CIGS/Mo (T)	1.01	34.11	81.66	28.4	67
CIGS (cell) (Cd-free) (E)	0.73	39.58	80.40	23.85	68
ZnO:Al/CdS/CIGS/Mo (E)	0.67	34.90	77.60	18.10	69
ZnO:Al/i-ZnO/CdS/CIGS/Mo (E)	0.73	32.00	—	17.50	66
ZnO/CdS/CuInGaSe_2_ (E)	0.69	35.50	81.20	19.80	70
ZnO/CdS/PDT-treated CIGS (E)	0.74	37.80	80.60	22.60	71
ZnO:B/i-ZnO/CdS/CIGS/Mo (E)	0.645	36.80	76.00	18.00	72
CIGS/GaAs (BSF)	1.16	43.88	89.52	45.7	73
CIGS/CNGS (BSF)	1.21	32.25	75.0	29.39	13
CIGS/V_2_O_5_ (BSF)	0.89	41.34	85.8	31.86	74
CIGS/CuO (BSF)	0.96	37.07	83.71	29.88	75
CIGS/Cu_2_O (BSF)	0.92	40.40	83.34	31.13	9
CIGS/Cu_2_O (BSF)	0.83	36.81	82.73	25.25	55
CIGS/Sb_2_S_3_ (BSF)	1.08	33.75	88.50	31.15	63
CIGS/CIGS-p^+^ (BSF)	1.14	32.61	89.39	33.36	22
CIGS/Cu_2_O (BSF)	0.86	40.85	86.69	30.30	54
CIGS/CuAlO_2_ (BSF)	0.82	35.87	83.11	24.61	7
CIGS/PEDOT:PSS (BSF)	0.86	56.40	80.79	32.83	76
CIGS/SWCNT (BSF)	0.80	42.59	80.38	27.73	8
CIGS/Sb_2_S_3_ (BSF)	0.760	29.84	81.66	37.05	In this study
CIGS/CuO (BSF)	0.660	30.27	79.47	31.76	In this study
CIGS/Cu_2_O (BSF)	0.815	30.30	82.88	40.34	In this study

**Fig. 17:**
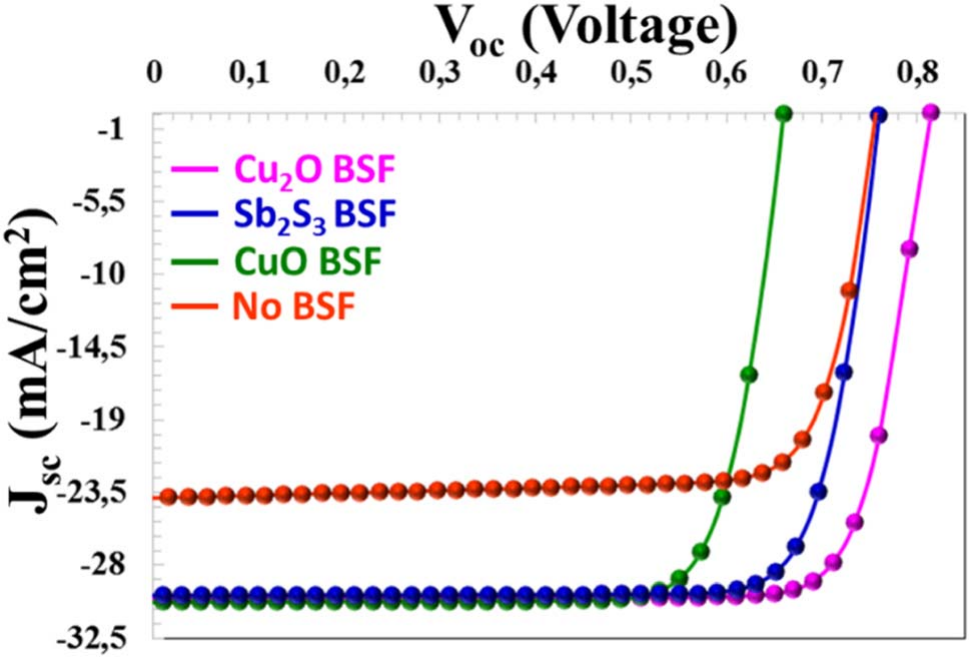
The dependence of *J*−*V* characteristics on BSF layer thickness for CIGSe solar cell without a BSF and with Sb_2_S_3_, CuO, and Cu_2_O BSF layers.

Increasing the thickness of the BSF layer can increase the p-type active layer doping differential, which can increase and stabilize the magnitude of the internal electric field formed on the back surface, forming a repulsive force against electrons (minority carriers).^15^ It is emphasized that passivating surface defects suppresses recombination by neutralizing active trap states and reinforcing the electric field at the rear junction. A thicker BSF layer enhances the separation between electrons and holes, suppressing recombination, and simultaneously facilitates better carrier alignment, leading to improved charge collection at the electrode. Increasing the thickness of BSF layers from 10 nm to 100 nm improves both charge transport and light absorption, resulting in a notable increase in efficiency. The Fig. 17 shows the *J*–*V* curve of a CIGSe solar cell modeled with Sb_2_S_3_, CuO, and Cu_2_O BSF layers. The *J*_sc_ values of the BSF solar cells are close to each other and high, while the *V*_oc_ value of the cell with CuO BSF is lower, and that of the structure with Cu_2_O BSF is higher. As a result, the highest solar cell efficiency was obtained by a Cu_2_O BSF-containing solar cell, according to the *J*−*V* characteristic shown in Fig. 17.

The simulated photovoltaic performance parameters obtained from SCAPS-based modeling, both in the present work and in previously reported studies, are comparatively overestimated. This discrepancy arises because SCAPS-1D, owing to its one-dimensional framework, cannot fully account for several critical physical and structural phenomena inherent to experimentally fabricated solar cells. Morphological properties, including lateral carrier transport, grain boundary effects, localized defect states, microcracks, pinhole formation, and surface roughness, are not explicitly accounted for, and all constituent layers are treated as compositionally uniform despite the presence of realistic compositional gradients and doping inhomogeneities in fabricated devices. Intricate defect landscapes and multi-level trapping mechanisms are reduced to simplified representations, while optical phenomena such as light scattering and wave–optical interactions, as well as mechanical stress and thermal coupling effects, are omitted from the model. At material interfaces, processes such as interdiffusion, interfacial chemical reactions, and phase formation are not explicitly resolved, with interface recombination instead approximated through effective defect density parameters. Likewise, non-idealities in metal/semiconductor contacts, including Fermi-level pinning, finite contact resistance, and interfacial roughness-induced transport barriers, are only implicitly treated or neglected. Consequently, the simulated photovoltaic device performance, whether modest or enhanced, remains within the range reported in the existing literature, demonstrating overall consistency with previously published results.

### 2.8. Capacitance–voltage (*C*–*V*) and Mott–Schottky characteristics

The junction's intrinsic properties critically govern the reproducibility of measurements, with the total p–n junction capacitance arising from the additive effects of both depletion and diffusion contributions. Depletion capacitance dominates under reverse bias, while diffusion capacitance prevails under forward bias. *C*–*V* characteristic presents the absorber and interfacial mechanisms, enabling precise evaluation of the doping concentration (*N*_a_) and built-in potential (*V*_bi_) through the subsequent relation:^46,77^15
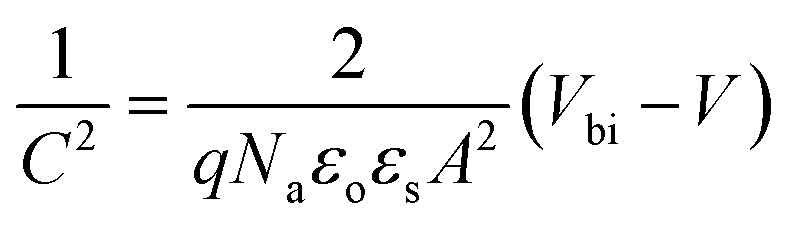
16
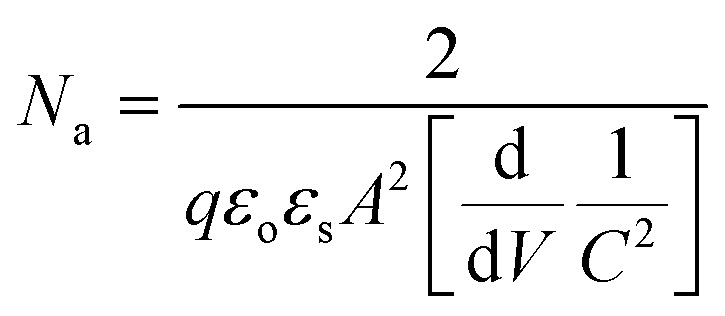
here, *N*_a_ denotes the doping concentration (cm^−3^), *q* represents the elementary charge (C), and *ε*_o_ and *ε*_s_ correspond to the vacuum permittivity (8.85 × 10^−14^ F cm^−1^) and the material's relative dielectric constant (from Table 1), respectively. *A* is the active area of the device, *C* is the capacitance, and *V* is the externally applied voltage.

**Fig. 18:**
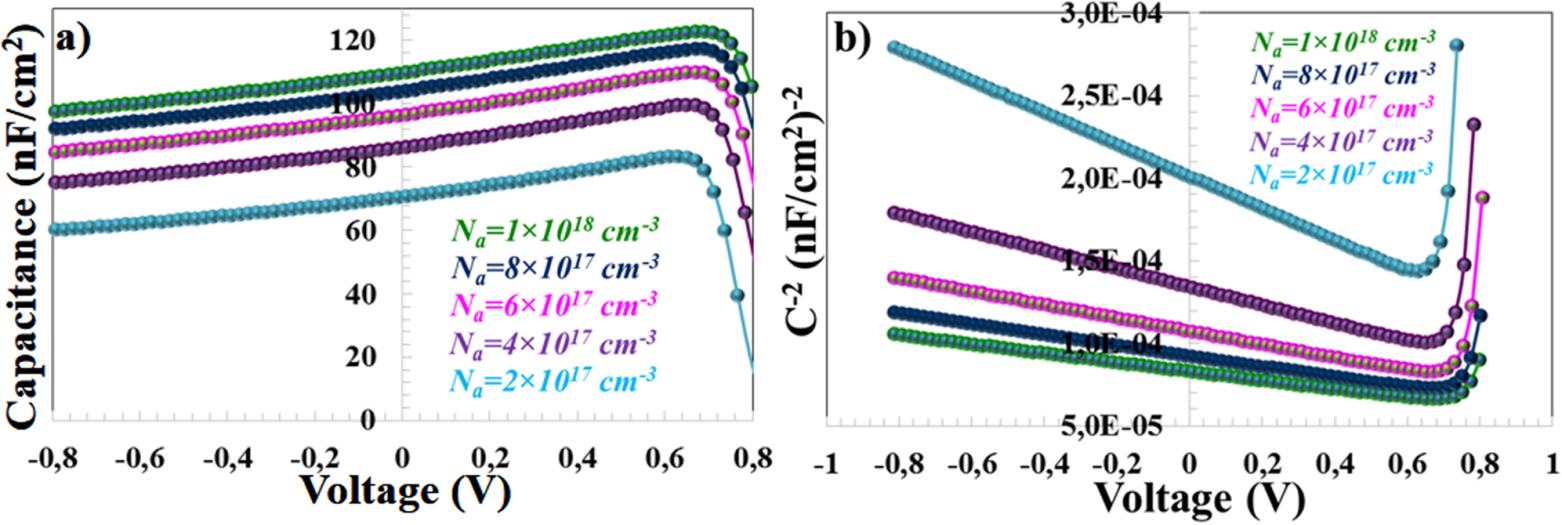
(a) *C*–*V* and (b) *C*^−2^−*V* characteristic depending on *N*_a_ variation.

**Fig. 19:**
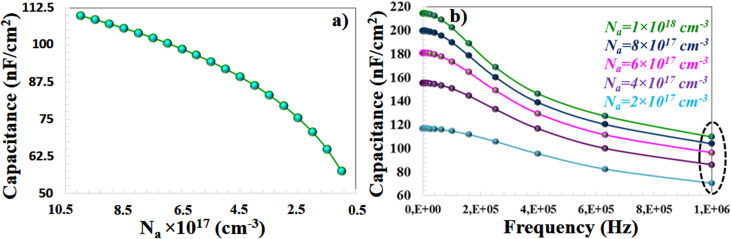
(a) Capacitance variation and (b) *C*−*f* curve depended on *N*_a_ value.

The Mott–Schottky analysis is widely recognized as a powerful and reliable method for extracting both the *V*_bi_ and the doping density of a device. The slope of the 1/*C*^2^–*V* relationship in a Mott–Schottky analysis reflects the concentration of electrically active trapping centers.^78,79^*C*–*V* and *M*–*S* analyses were simulated at a frequency of 10^6^ MHz. Fig. 18 presents a detailed examination of (*C*–*V*) behavior alongside the Mott–Schottky analysis for the proposed solar cell, highlighting the influence of the shallow, uniformly distributed acceptor concentration (*N*_a_) within the CIGSe absorber layer. The acceptor density (*N*_a_) was systematically adjusted across a range spanning 10^17^ to 10^18^ cm^−3^. As illustrated in Fig. 18(a), the device capacitance increases progressively with increasing applied bias, ultimately reaching a maximum at elevated voltages. Fig. 18(a) shows that the device is fully depleted at zero applied bias. The capacitance value, dependent on *N*_a_, at *V* = 0 is shown in Fig. 19(a). When *N*_a_ is increased from 10^17^ to 10^18^ cm^−3^, the capacitance value increases from 57.57 to 109.94 nF cm^−2^. Consequently, applying an increasing forward bias gradually increases the capacitance, reflecting the progressive modulation of the depletion region. An increase in doping levels promotes charge buildup at the interface, thereby increasing capacitance. The Mott–Schottky analysis indicates that the built-in potential (*V*_bi_) can be extracted from the intercept at 1/*C*^2^ = 0 on the potential axis in Fig. 18(b). *V*_bi_ values for *N*_a_ = 1 × 10^18^, 8 × 10^17^, 6 × 10^17^, 4 × 10^17^, and 2 × 10^18^, which have been determined to be 0.80, 0.88, 0.96, 1.20, 1.32 eV, respectively. The increase in *V*_bi_ indicates a more effective internal electric field, facilitating charge separation.

The analysis of *C*−*f* characteristics provide valuable insight into the device's electronic behavior and deep defect states, as well as the impact of charge migration on frequency-dependent capacitance and conductance.^46,78^ AC signal changes slowly, at low frequencies. Electrons and holes (especially minority carriers) readily follow the signal, and more charge accumulates in the p–n heterojunction. Minority-carrier injection increases, raising the charge density in the junction. However, at high frequencies, the signal changes very rapidly. Electrons and holes cannot keep up with this rapid change. In particular, the minority carrier contribution disappears. Deep defects require time to receive and release charge. Diffusion and injection contributions are lost. Charge accumulation within the p–n junction is limited. This process cannot occur at high frequencies. It is observed that the capacitance values at *V* = 0 in Fig. 18(a) correspond to those measured at 1 MHz in Fig. 19b.^46^

## 3 Conclusions

In this work, a comprehensive SCAPS-1D simulation of an ultra-thin (420 nm) CIGSe/CdS/i-ZnO/ITO solar cell structure incorporating various p^+^-type BSF layers (Sb_2_S_3_, CuO, and Cu_2_O) has been systematically performed. The results clearly demonstrate that device performance is highly sensitive to key physical and defect-related parameters. An increase in the acceptor defect density (*N*_a_) from 10^13^ to 10^18^ cm^−3^ significantly enhances the PV response, yielding improvements in *V*_oc_ (from 0.298 to 0.757 V), FF (from 26.78% to 55.97%), and efficiency (from 3.83% to 19.95%), primarily due to improved carrier concentration and stronger internal electric fields.

Conversely, higher interface defect density (*N*_i–t_ > 10^13^ cm^−3^) and bulk defect density (*N*_t_ > 10^16^ cm^−3^) result in severe recombination losses, drastically reducing device performance. Optimal band alignment was achieved at an electron affinity of *χ* = 4.35 eV, corresponding to a slight negative conduction-band offset (Δ*E*_c_ = −0.05 eV), which minimizes recombination at the heterointerface. Recombination analysis further revealed that radiative recombination remains negligible within 10^−16^ to 10^−8^ cm^3^ s^−1^, while Auger recombination becomes dominant beyond 10^−23^ cm^6^ s^−1^, leading to rapid efficiency degradation. Additionally, reducing series resistance (from 10 to 1 Ω cm^2^) and increasing shunt resistance (from 10^1^ to 10^4^ Ω cm^2^) further improved charge collection and minimized leakage losses. Furthermore, capacitance–voltage (*C*–*V*) and Mott–Schottky analyses revealed that capacitance increases with *N*_a_ (from 57.57 to 109.94 nF cm^−2^ at *V* = 0), indicating enhanced charge accumulation and modulation of the depletion region. The extracted built-in potential (*V*_bi_) ranged from 0.80 to 1.32 eV, confirming that the internal electric field strength increases with higher doping levels. Among the investigated BSF layers, Cu_2_O exhibited superior performance due to its wider band gap (2.2 eV) and enhanced back surface electric field, achieving a maximum efficiency of 40.34% with *V*_oc_ = 0.817 V, *J*_sc_ = 30.03 mA cm^−2^, and FF = 82.88%.

## Conflicts of interest

The authors declare that they have no known competing financial interests or personal relationships that could have influenced the work reported in this paper.

## Data Availability

The data supporting this study's findings are available from the corresponding author upon reasonable request.
